# Relation between Mood and the Host-Microbiome Co-Metabolite 3-Indoxylsulfate: Results from the Observational Prospective NutriNet-Santé Study

**DOI:** 10.3390/microorganisms9040716

**Published:** 2021-03-31

**Authors:** Catherine Philippe, Fabien Szabo de Edelenyi, Laurent Naudon, Nathalie Druesne-Pecollo, Serge Hercberg, Emmanuelle Kesse-Guyot, Paule Latino-Martel, Pilar Galan, Sylvie Rabot

**Affiliations:** 1 Micalis Institute, INRAE, AgroParisTech, Université Paris-Saclay, 78350 Jouy-en-Josas, France; 2 Sorbonne Paris Nord, Epidemiology and Statistics Research Center (CRESS), Inserm U1153, INRAE U1125, Cnam, Paris 13 University, Nutritional Epidemiology Research Team (EREN), 93017 Bobigny, France; n.pecollo@eren.smbh.univ-paris13.fr (N.D.-P.); s.hercberg@eren.smbh.univ-paris13.fr (S.H.); e.kesse@eren.smbh.univ-paris13.fr (E.K.-G.); paule.latino-martel@inra.fr (P.L.-M.); 3 INRAE, AgroParisTech, CNRS, Micalis Institute, Université Paris-Saclay, 78350 Jouy-en-Josas, France; laurent.naudon@inrae.fr

**Keywords:** tryptophan, indole, 3-indoxylsulfate, gut microbiota, mood disorders, depression, biomarker, Nutrinet-Santé Study, female subjects

## Abstract

Gut microbiota metabolizes tryptophan into indole, which can influence brain and behavior. Indeed, some oxidized derivatives of indole, formed in the liver, have neuroactive properties, and indole overproduction by the gut microbiota induces an anxio-depressive phenotype in rodents. The aim of this study was to investigate in humans whether there was a relationship between recurrent depressive symptoms and indole production by the gut microbiota. A case-control study was conducted in 45–65-year-old women, who were participants in the observational prospective NutriNet-Santé Study. Cases were defined as having two Center for Epidemiological Studies-Depression Scales (CES-D) scores ≥ 23 at a two-year interval (recurrent depressive symptoms, *n* = 87). Each case was matched with two controls (two CES-D <23; *n* = 174). Urinary excretion of 3-indoxylsulfate, the major final metabolite of indole, was used as a biomarker of indole production by the gut microbiota. Conditional logistic regression models for paired data showed a positive association between urinary 3-indoxylsulfate concentrations, grouped in tertiles, and recurrent depressive symptoms (odds ratio = 2.46, *p* for trend = 0.0264 in the final model adjusted for confounding factors). This association suggested that indole production by the gut microbiota may play a role in the onset of mood disorders in humans.

## 1. Introduction

A large number of studies in laboratory animals definitively demonstrated the existence of a crosstalk between the gut microbiota and the brain, leading to the concept of the gut microbiome–brain axis [1]. On this basis, the challenges today are (i) to characterize the microbial actors, the communication pathways they use to signal to the brain, and the mechanisms by which they interact with specific brain structures, (ii) and to identify to which extent the observations made in laboratory animals can translate to humans [2].

Among the microbial actors, metabolites resulting from the catabolism of aromatic amino acids are suspected to play an important role. Comparative analysis of the plasma metabolome of germ-free and conventional mice indicated that the microbiota enriches the plasma with metabolites derived from tryptophan, phenylalanine, and tyrosine [3]. Hsiao et al. [4] showed in a mouse model simulating behavioral abnormalities of autism spectrum disorders that the gut microbiota composition was modified; this modification was accompanied by an enrichment of the plasma with a host-microbiome co-metabolite derived from tyrosine, the 4-ethylphenylsulfate. Systemic administration of this molecule to naïve mice induced anxiety-like behavior. Interestingly, *p*-cresol, a microbial metabolite chemically related to 4-ethylphenylsulfate, was reported as a possible urinary biomarker for autism spectrum disorders [5]. Moreover, in humans with mild to moderate anxiety and/or depression, the probiotic *Bifidobacterium longum* NCC3001 reduced the urinary excretion of several host-microbiome co-metabolites derived from aromatic amino acids; among them, *p*-cresol sulfate correlated positively with depression scores [6].

We showed previously in gnotobiotic rodent models that indole produced by microbial hydrolysis of tryptophan in the large intestine intensified the anxiety-like and depressive-like phenotypes, as assessed by several behavioral tests [7,8]. The gut microbiota hydrolyses tryptophan into indole through the action of the enzyme tryptophanase (TnaA proteins) [9]. By analyzing in silico a human metagenomic catalogue, we showed that the *tna*A genes encoding the TnaA proteins are widely distributed in the gut microbiome, but that the *tnaA* gene richness varies greatly, depending on the individual [7]. This indicated that some people have a gut microbiome with a high potential for indole production, while others have a gut microbiome with a low potential. On those bases, it can be hypothesized that mood in humans could be related to the ability of the gut microbiota to produce indole.

**Figure 1:**
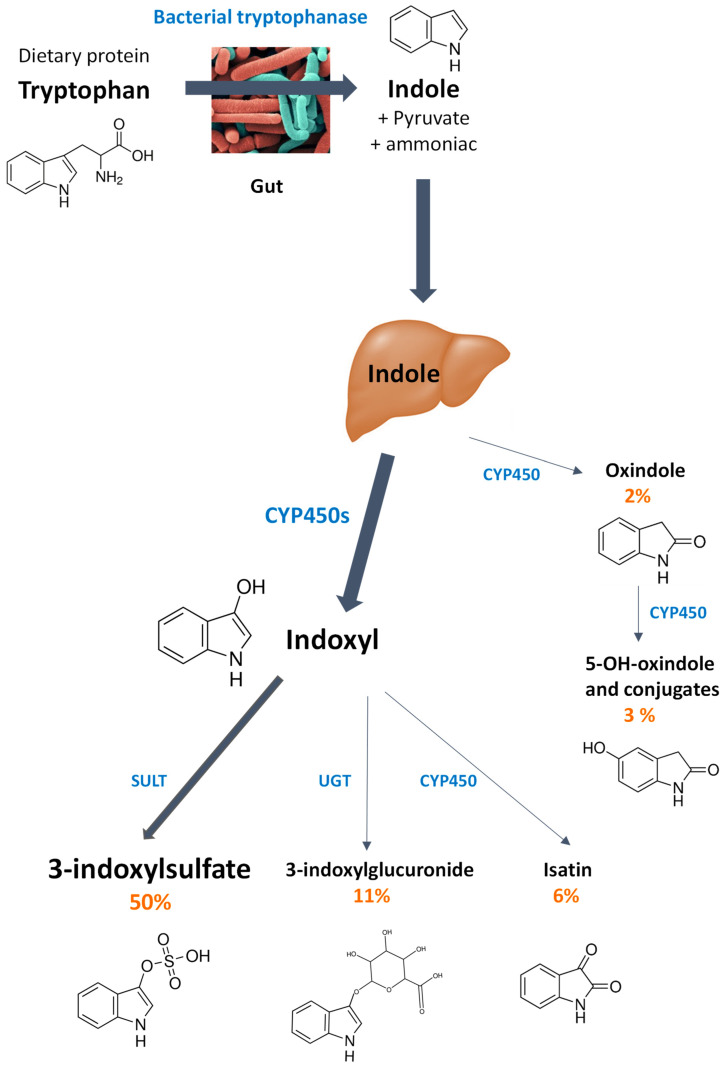
Metabolites of indole excreted in urine. Indole is produced from dietary tryptophan by the gut microbiota tryptophanase [9]. Then, indole is absorbed and metabolized in the liver by different cytochrome P450 isozymes (CYP450s), arylsulfotransferases (SULT), and UDP-glucuronosyltransferases (UGT) [12,13,14]. Experiments in which an oral bolus of [2-^14^C] indole was given to rats showed that 80% of the radioactivity was excreted in urine. 3-Indoxylsulfate was the major metabolite (50% of the ingested dose). Other minor metabolites, namely 3-indoxylglucuronide, isatin, oxindole, and 5-OH-oxindole and conjugates were identified and accounted for 11, 6, 3, and 2% of the ingested dose, respectively [15].

The aim of this study was to test this hypothesis in the observational prospective NutriNet-Santé Study carried out in France [10,11]. Participants in this study answer questionnaires on their dietary habits, lifestyle characteristics, physical activity, and health events on a regular basis over a ten-year follow-up period. In particular, a mood self-assessment questionnaires were administered twice at a two-year interval, making it possible to estimate the existence of recurrent depressive symptoms. Biological samples were also taken from some of the participants. They included blood and urine, but not stools. This made it impossible to analyze the gut microbiota *tna*A gene richness and indole production. To overcome this issue, urinary 3-indoxylsulfate was chosen as a biomarker of the production of indole by the gut microbiota. Indeed, once excreted by bacterial cells in the gut lumen, indole is absorbed and metabolized in the liver by xenobiotic metabolizing enzymes into a family of oxidized and conjugated derivatives, including 3-indoxylsulfate [12,13,14,15] (Figure 1). King et al. showed in rats that 80% of the radioactivity of an oral bolus of [2-^14^C] indole was recovered in urine within 2 days and that 3-indoxylsulfate accounted for 70% of this excretion, i.e., 50% of the ingested dose [15]. Other, minor metabolites were also identified but, overall, they accounted only for 22% of the ingested dose. In addition, 3-indoxylsulfate is strictly related to the production of indole by the gut microbiota. Indeed, indole and 3-indoxylsulfate are consistently absent from the feces and plasma of germ-free rodents, respectively [3,16]. In addition, gnotobiotic rodents with a gut microbiota producing no indole excrete no 3-indoxylsulfate in urine, while they do when their gut microbiota produce indole [7,8]. Based on those findings, 3-indoxylsulfate can be considered as a host-microbiome co-metabolite, whose concentration in urine reflects the microbial production of indole in the gut. Therefore, in this study, the research focused on the relationship between 3-indoxylsulfate urinary excretion and recurrent depressive symptoms.

In a companion study carried out with another population sample of the NutriNet-Santé Study, and focusing on the relationships between diet and recurrent depressive symptoms, and diet and 3-indoxylsulfate [17], no significant correlation was found between recurrent depressive symptoms and 3-indoxylsulfate. However, the cut-off to define depressive symptoms in the mood questionnaire was set at the internationally defined level. In the present study, the cut-off validated specifically for the French population [18,19] was applied.

## 2. Materials and Methods

### 2.1. Population

Subjects were selected among participants of the NutriNet-Santé Study, which was a large observational prospective cohort study including adult volunteers at least 18 years of age. The NutriNet-Santé Study was launched in France in May 2009 with a scheduled follow-up of 10 years. It was designed to study the relations between nutrition and health and its protocol was described in details elsewhere [10]. Briefly, at baseline, participants completed a set of questionnaires assessing anthropometry, socioeconomic and health status, dietary intake, physical activity, and lifestyle. Afterwards, they were invited regularly to fill in non-compulsory complementary questionnaires related to determinants of eating behaviors, nutritional and health status. Among them, the French version of the Center for Epidemiological Studies-Depression Scale (CES-D) was used to assess depressive symptoms [18,19]. It was proposed to the participants at 26 and 53 months after their inclusion. All questionnaires were web-based and self-administered. The NutriNet-Santé Study was conducted according to the guidelines in the Declaration of Helsinki. It was approved by the ethics committee of the French Institute for Health and Medical Research (IRB Inserm n° 0000388FWA00005831) and by the National Commission of Informatics and Liberty (CNIL n° 908450 and n° 909216). It is registered in ClinicalTrials.gov (NCT03335644).

Participants were also invited, on a voluntary basis, to a visit to a dedicated center in a hospital close to their home, for a clinical examination and biological sampling (blood, urine). Those attending the visit signed an electronic and paper consent. All the procedures were approved by the “Consultation Committee for the protection of Participants in Biomedical Research” (C09-42 on 5 May 2010) and the National Commission of Informatics and Liberty (CNIL n° 1460707).

### 2.2. Selection of the Study Sample

**Figure 2:**
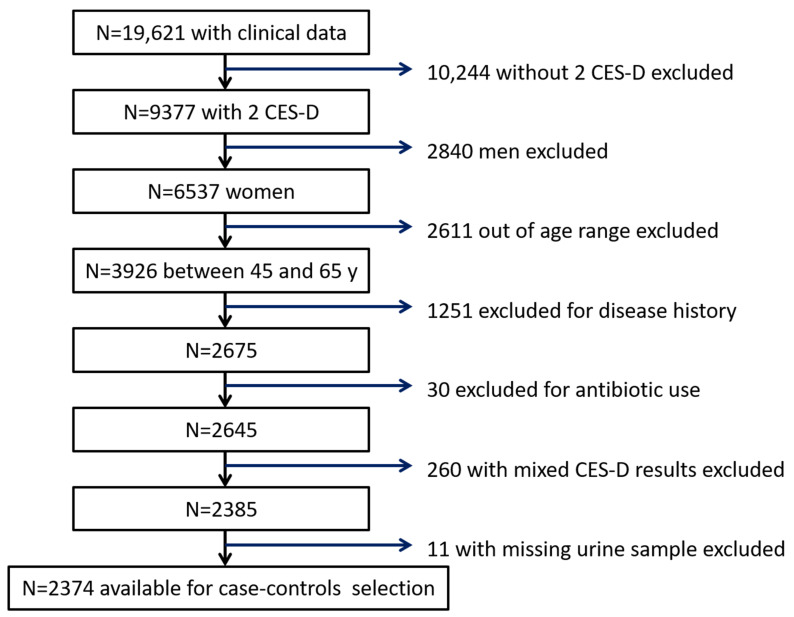
Flow chart of participants’ selection. CES-D: Center for Epidemiologic Studies-Depression Scale.

Depressive episodes are twice as common in women as in men and increase with age [20]. Therefore, to reduce statistical heterogeneity due to age and gender, we selected 3926 women aged 45 to 65 years at clinical examination, who had filled out the CES-D questionnaire twice at 26 and 53 months after inclusion, and for whom biological samples and at least three dietary records were available. The following criteria were also taken into account: no declaration of history (prevalent or incident) of gastrointestinal disease (hepatitis, Crohn disease, haemorrhagic rectocolitis, gluten intolerance, colopathy or irritable bowel syndrome), cancer, myocardial infarction, rheumatoid arthritis, sarcoidosis, multiple sclerosis, lupus erythematosus or ankylosing spondylitis; no antibiotic use at the time of biological sampling. After applying these exclusion criteria, there were 2374 participants available for case-control selection (Figure 2).

The case-control sample finally included 261 subjects. The CES-D cut-off score validated for the French population to define the presence of recurrent depressive symptoms was applied [18,19]. Consequently, cases were defined by CES-D scores ≥ 23 both at 26 and 53 months after inclusion (*n* = 87), while controls were defined by two CES-D scores < 23 (*n* = 174).

Each case was matched with two controls on the following parameters: age (age at clinical examination, within a 5-year interval between cases and controls); employment status (without activity, in activity, retired); socio-professional category (managerial staff, intermediate professions, manual worker + employee + farmer) [21]. The category of intermediate professions was made up of professions such as teachers, nurses, and social workers for 1/3, and professions in which individuals have an intermediate position between manual workers + employees and managerial staff for 2/3. These variables were chosen because depression has been associated with age, employment status, and income in France [19]. However, as many income data were missing, socio-professional categories were used instead.

### 2.3. Data Collection

#### 2.3.1. Depressive Symptoms

Depressive symptoms were assessed with the self-administered CES-D questionnaire, which is widely used for the assessment of depressive symptoms at a population level. It included 20 items that evaluate the frequency of symptoms and behavioral characteristics associated with depression during the previous week, using a four-point scale (0 = “less than a day”; 1 = “1–2 days”; 2 = “3–4 days”; 3 = “5–7days”). The responses were added to yield a total score between 0 and 60, the higher scores reflecting more depressive symptoms.

#### 2.3.2. Analysis of 3‑Indoxylsulfate in Urine

3‑Indoxylsulfate was analyzed by HPLC with fluorescence detection, using the method of Deguchi et al. [22] with slight modifications. Urine samples were thawed and clarified by centrifugation (12,000× *g*, 15 min, 4 °C). The supernatants were 20-fold diluted in acetate buffer (50 mM, pH 4.0) and 50-µL samples were injected onto a Kinetex^®^ reversed-phase C18 column (5 µm, 250 mm × 4.6 mm; Phenomenex, Le Pecq, France), equipped with a security guard ULTRA cartridge UHPLC C18 (Phenomenex), using a 2690-autosampler and separation module (Waters). Elution was isocratic (25% acetonitrile and 75% 50-mM acetate buffer pH 4.0) at a flow rate of 1 mL/min. The excitation and emission wavelengths of the 474-fluorescence detector (Waters) were set at 280 nm and 375 nm, respectively. 3‑Indoxylsulfate eluted at 6.9 min; it was quantified using an external standard curve. Analyses were carried out in duplicate. All calculations were performed with the Millenium^®^ software (Waters, Milford, MA, USA).

Urinary 3-indoxylsulfate concentrations were expressed relatively to urinary creatinine concentrations. Creatinine was measured in duplicate using a colorimetric kit according to the manufacturer’s instructions (Enzo, Villeurbanne, France).

#### 2.3.3. Dietary Data

Dietary data were collected at baseline, then every 6 months, in the form of 3 dietary records over 24 h (2 records on weekdays and 1 record on a weekend day), randomly distributed over a 2-week period. Participants reported all foods and beverages consumed throughout the day. Serving sizes were estimated using purchase unit, household unit, and photographs, derived from a previously validated booklet [23]. Alcohol consumption was calculated from 24-h dietary records, according to a food composition database [24], and with a weighting on the type of day (weekday or weekend day).

#### 2.3.4. Other Descriptive Characteristics

Other descriptive characteristics, some of which were used as covariates, were collected at baseline through self-administered questionnaires. They provided information on birth date, educational level (primary or no diploma, secondary, post-secondary), marital status (living alone, i.e., single, divorced or widow; cohabiting, i.e., married or non-married couple), smoking status (never, former, or current smoker), occupational category, body mass index, leisure time physical activity, and season of inclusion. Occupational category was defined as a combination of socio-professional category and employment status (socio-professional category within each employment status). Body mass index was calculated using self-reported height and weight and was used to define three corpulence classes (<25 kg/m^2^, 25–30 kg/m^2^, ≥30 kg/m^2^). Leisure time physical activity was assessed using the French short form of the International Physical Activity Questionnaire (IPAQ). The metabolic equivalent (MET) measured in min/week was computed. Recommended IPAQ categories of physical activity (low, i.e., < 30 min brisk walking/day; moderate, i.e., 30–60 min/day; and high, i.e., ≥60 min/day) were used in the analyses [25,26,27]. An “unknown” category was added for this variable, as many participants did not answer the question (missing data). The season of inclusion in the NutriNet-Santé cohort was defined as follows: fall (September to November), winter (December to February), spring (March to May), summer (June to August). This variable was taken into account as season may affect mood and depressive symptoms, and hence, responses to the CES-D questionnaire [28].

### 2.4. Statistical Analyses

Descriptive characteristics of controls and cases are presented as means (SD), medians [interquartile range] or percentages. Distributions of the urinary 3-indoxylsulfate concentrations (expressed as µmol/mg creatinine) in controls and cases are shown in a box plot. *p* values refer to conditional logistic regression models for matched data.

The relation between the urinary 3-indoxylsulfate concentrations and the CES-D scores was analyzed in two steps. First, a linear regression was performed between the two parameters and the correlation coefficient was calculated. Second, conditional logistic regression models for paired data were applied to analyze the association between the urinary concentrations of 3-indoxylsulfate and recurrent depressive symptoms (two CES-D scores ≥ 23), without and with adjustment for potential confounding factors. For this, 3-indoxylsulfate concentrations (expressed as µmol/mg creatinine) were log_10_-transformed to make data conform to normality and grouped in tertiles. Model 1 was crude. Model 2 was adjusted for age. Model 3 added adjustment for educational level (primary or no diploma, secondary, post-secondary) and marital status (living alone or cohabiting). Model 4 added adjustment for alcohol consumption, smoking status (never, former, or current smoker), physical activity (low, moderate, high, or unknown), and season of inclusion in the NutriNet-Santé Study. Model 5 added body mass index (<25 kg/m^2^, 25–30 kg/m^2^, ≥30 kg/m^2^) as a covariate. This analysis provided odds ratio (OR) and 95% confidence interval (CI). *p* for trend was obtained from modelling tertiles as an ordinal variable in the model.

In addition, the association between urinary concentrations of 3‑indoxylsulfate and fruit and vegetable intake was studied using multivariate analysis of variance models. General linear models with log_10_ [3‑indoxylsulfate/creatinine] as outcome and tertiles of fruit and vegetable intake as dependent variable were applied, without and with adjustment for the same potential confounding factors as those cited above.

All analyses were carried out using SAS (version 9.4; SAS institute Inc., Cary, NC, USA) with a significance level of 0.05 for two-sided tests.

## 3. Results

Table 1 shows the descriptive characteristics of controls (*n* = 174) and cases (*n* = 87). Women with recurrent depressive symptoms lived more alone than women of the control group (35.6% versus 18.4%; *p* = 0.004). The percentage of obese women (BMI ≥ 30) was greater in the case group than in the control group (21.8% versus 6.3%; *p* = 0.003), but there were fewer overweight women in the case group compared to the control group (18.4% versus 25.9%; *p* = 0.003). There was no significant difference between cases and controls for the other descriptive characteristics.

**Figure 3:**
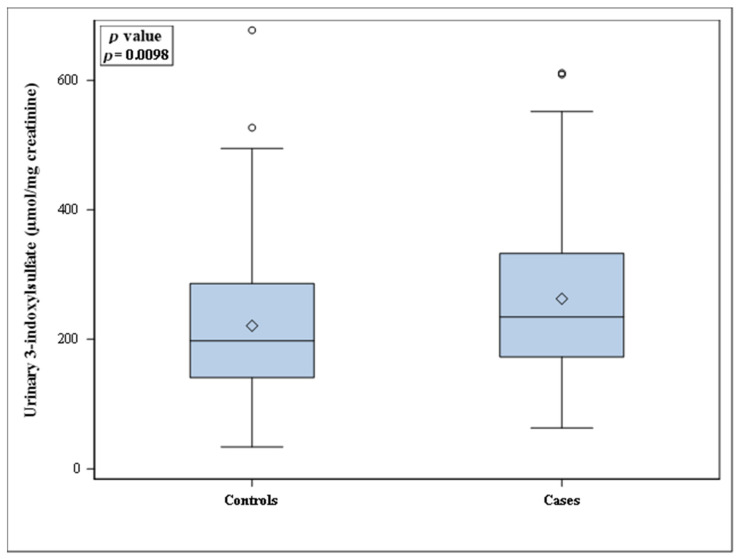
Box plot of urinary 3-indoxylsulfate concentrations (expressed as µmol/mg creatinine) in controls (*n* = 174) and cases (*n* = 87). Open diamonds inside the boxes are means; open circles outside the boxes are outliers.

**Figure 4:**
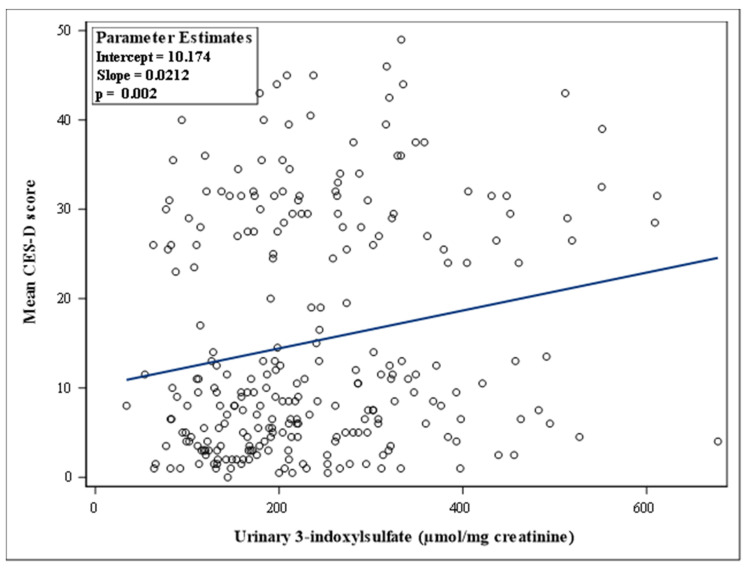
Scatter plot of the mean values of the two CES-D scores per individual (*n* = 261) versus urinary 3-indoxylsulfate concentrations (expressed as µ mol/mg creatinine).

Figure 3 shows the distribution of urinary 3-indoxylsulfate concentrations, expressed as µmol/mg creatinine. These concentrations were greater in the case group than in the control group: medians [interquartile range] were 234.2 [172.6–332.6] and 197.3 [140.6–286.0] in the case and control groups, respectively (*p* = 0.0098). Furthermore, these concentrations were positively correlated with the CES-D scores (r = 0.1908; *p* = 0.002), as shown in Figure 4.

Investigation of the association between urinary concentrations of 3-indoxylsulfate and recurrent depressive symptoms was refined by using conditional logistic regression models, crude and adjusted for potential confounding factors. In these models, 3-indoxylsulfate concentrations were log_10_-transformed to make data conform to normality (means ± SD were 2.29 ± 0.21 and 2.36 ± 0.23 in controls and cases, respectively) and grouped in tertiles. The association was significantly positive in the crude model (*p* for trend: 0.0088) and remained significant after adjustment for confounding factors (*p* for trend from 0.0066 to 0.0264) (Table 2). In all models, women in the third tertile of 3-indoxylsulfate concentrations had a higher risk to have recurrent depressive symptoms (OR between 2.38 and 2.65).

A significantly negative association between fruit and vegetable consumption and urinary concentrations of 3-indoxylsulfate was found in our companion study [17]. Therefore, this association was also tested in the current study, using general linear models with urinary concentrations of 3-indoxylsulfate as outcome and tertiles of fruit and vegetable intake as dependent variable. The association was significantly negative in the crude model (*p* for trend: 0.0038) and remained significant after adjustment for confounding factors (*p* for trend from 0.0011 to 0.0023) (Table 3).

## 4. Discussion

In this case-control study focused on women aged 45–65 years, those with recurrent depressive symptoms were more likely to be obese. This result was in line with the findings of meta-analyses studying the relationships between depression and obesity. A meta-analysis including 17 cross-sectional studies concluded to a significant positive association between depression and obesity in the general adult population, and this association was more marked in women [29]. Furthermore, a meta-analysis including 15 longitudinal studies showed that obesity at baseline increased the risk of onset of depressive symptoms in adults [30]. Conversely, in the same population, baseline depressive symptoms increased the risk of developing obesity [30]. In the current study, women with recurrent depressive symptoms were also more likely to live alone. The association between marital status and depression has been well documented. By analyzing depression data from 18 countries, Bromet et al. [20] showed a strong association of being separated or never married with depression in high-income countries, such as France. Analysis from data of a longitudinal health survey in Canada showed that depression increases the risk of marital disruption; conversely, separated or divorced status increases the risk of depression [31].

Women with recurrent depressive symptoms also had a higher urinary excretion of 3-indoxylsulfate. The conditional logistic regression model used to examine the association between those two parameters showed that being in the upper tertile of 3-indoxylsulfate excretion was significantly associated with recurrent depressive symptoms (odds ratio > 2.0). It should be emphasized that this association remained significant even after controlling for confounding factors including body mass index and marital status. Differences in the urinary excretion of the host-microbiome co-metabolite 3-indoxylsulfate may reflect differences in indole production by the gut microbiota, which, in turn, may be due to differences in microbiota composition. Cases may have larger populations of bacterial cells with a *tna*A gene that encodes the enzyme tryptophanase, which is responsible for the hydrolysis of tryptophan into indole [9] (Figure 1). The in silico analysis of a human gut metagenomics catalog we carried out previously allowed to identify nearly 400 bacterial genes putatively coding for tryptophanases (i.e., *tna*A-like genes). A third of them only could be assigned to known species, mainly belonging to the phyla *Bacteroidetes* (*Bacteroidaceae* and *Rikenellaceae*), *Firmicutes* (*Clostridiaceae* and *Lachnospiraceae*), and *Proteobacteria* (*Enterobacteriaceae* and *Rhodobacteraceae*). Distribution of those *tna*A-like genes in a panel of 200 individuals showed substantial inter-individual variation, with the richness of non-redundant *tna*A-like genes ranging from 5 to 100 [7]. As there was no fecal sample collection in the NutriNet-Santé Study, the gut microbiota composition of the women included in the study sample could not be analyzed. However, different studies comparing the composition of the gut microbiota of depressed patients and healthy subjects systematically revealed differences [32,33,34,35,36,37]. Although there is no consensus on the observed changes for the moment, it is interesting to note that two studies found that the *Alistipes* genus (phylum: *Bacteroidetes*, family: *Rikenellaceae*), whose most members are able to hydrolyze tryptophan into indole [38,39], was overrepresented in patients [32,33]. Likewise, mice subjected to a chronic stress procedure saw their gut microbiota evolve, with an increase of the *Alistipes* genus population [40]. On the other hand, Zheng et al. [35] found an overrepresentation of the *Alistipes* genus in healthy controls, compared to depressed patients. Overall, these data encourage to investigate the microbiota differences between controls and patients in terms of metabolic functionalities. In particular, it would be highly interesting to determine the prevalence of the *tnaA*-like genes and, more generally, of the genes encoding enzymes responsible for the degradation of aromatic amino acids, in patients with mood disorders vs. healthy subjects. Alternatively, the increased 3-indoxylsulfate excretion in the women with recurrent depressive symptoms may be related to dietary characteristics. Indeed, as in the companion study carried out with another women population of the NutriNet Santé study [17], urinary 3-indoxylsulfate excretion was inversely associated with fruit and vegetable consumption in the current study. Such findings were in agreement with those reported by Patel et al. [41] in a small group of vegetarians and omnivores. Indeed, the vegetarians excreted two-fold less 3-indoxylsulfate than omnivores. This difference was probably due to a higher dietary fiber intake in vegetarians. Increasing fiber intake increases substrate for microbial fermentation so that amino acids are consumed in microbial growth rather than broken down to produce energy. Interestingly, Patel et al. [41] observed that the excretion rate of 3-indoxylsulfate varied widely among individuals, even within a group with the same dietary pattern (vegetarian vs. omnivorous), but tended to remain stable within individuals over time. This suggested the existence of individual microbiota characterized by different potentials to produce indole as a function of their richness in *tna*A-like genes [7], but whose indole production can be modulated by the diet [42]. The association between indole production by the gut microbiota and recurrent depressive symptoms is supported by experimental evidence. A chronic overproduction of indole by the gut microbiota promotes anxiety- and depressive-like behaviors in rodents [7,8]. Indole may act on the brain through the vagus nerve, as suggested by the neuronal activation of the hindbrain area receiving signals from this nerve, namely the dorsal vagal complex, in rats overproducing indole [7]. In addition, host-microbiome co-metabolites of indole, such as isatin and oxindole (Figure 1), could be involved as they have repeatedly been shown to be neurodepressant molecules [43,44,45].

Our study had many strengths. It was based on a well characterized population in terms of health, dietary habits, and lifestyle, followed over a long period of time. Results from two CES-D questionnaires at a two-year interval were used to focus on chronic poor mood rather than on temporary depressive episodes. The regression models were adjusted for confounding factors, including marital status and body mass index, which are known to be related to depression and were indeed different between cases and controls. But it also had limitations. As stool samples were not available for microbiota and indole analyses, we had to turn to the analysis of a surrogate, the host-microbiome co-metabolite 3-indoxylsulfate, which is excreted in urine. Though it was clearly demonstrated that 3-indoxylsulfate is the major end product of indole produced by the gut microbiota [15], its production depends on the cytochrome P450 and arylsulfotransferase liver enzymes, which exhibit genetic polymorphisms [12,13,14,46,47]. Therefore, possible inter-individual differences in the extent to which indole was transformed into 3-indoxylsulfate cannot be ruled out in the study population. Besides, while some host-microbiome co-metabolites of indole, such as oxindole and isatin, have neurodepressant properties [43,44,45], other tryptophan derivatives produced by the gut microbiota, such as indole-propionic acid, were shown to reduce brain inflammation by modulating microglia and astrocyte activity [48,49]. This illustrates the fact that the same chemical family of gut microbiota metabolites can have beneficial or harmful effects on the brain. The overall result is likely to be driven by the profile and the quantity of these metabolites, which depend on gut microbiota composition and dietary habits, and by the physiological and environmental context of the host. This might explain why the current results are not in line with some other studies. For example, a negative association was found between plasma concentrations of 3-indoxylsulfate and type D personality, which reflected a “general propensity to psychological distress”, in a German cohort comprising male and female subjects aged between 32 and 77 years [50]. In a study comparing the urinary metabolome of Chinese male and female healthy subjects and patients with moderate or severe depression, whose age averaged 31–34 years, Chen et al. [51] reported a lower urinary concentration of 3-indoxylsulfate in patients with severe depression compared with healthy controls.

In this context, future studies in humans should involve metagenomics and metabolomics analyses aimed at characterizing the balance of the tryptophan metabolic pathways carried out by the gut microbiota in various groups of individuals, from those with poor mood to those clinically diagnosed with different levels of depression severity and response to treatment. Considering the links between diet and mood, those studies should also include dietary questionnaires. Together with mechanistic studies on the effects of tryptophan microbial derivatives on the nervous system, results of such epidemiological studies could help to provide novel bases for taking into account the gut microbiota in the therapeutic management of mood disorders.

## Figures and Tables

**Table 1:** Descriptive characteristics of cases and controls, with *p*-values for paired comparisons.

		Controls	Cases	*p*-Value ^1^
N		174	87	
Age (years)		55.8	55.7	0.76
(mean (SD))		(5.9)	(5.8)	
Age classes (%)	45–50	23.0	23.0	1 *
	51–55	23.0	23.0	
	56–60	25.3	25.3	
	61–65	28.7	28.7	
Educational level (%)	Primary or no diploma	1.1	6.9	0.08
	Secondary	45.4	43.7	
	Post-secondary	53.5	49.4	
Marital status (%)	Living alone	18.4	35.6	0.004
	Cohabiting	81.6	64.4	
Employment status and socio-professional category (%)	Without activity	20.7	20.7	1 *
	including:			
	• managerial staff	3.5	3.5	
	• intermediate professions	4.6	4.6	
	• farmers, employees, manual workers	12.6	12.6	
	In activity	62.1	62.1	
	including:			
	• managerial staff	13.8	13.8	
	• intermediate professions	19.6	19.6	
	• farmers, employees, manual workers	28.7	28.7	
	Retired	17.2	17.2	
	including:			
	• managerial staff	2.3	2.3	
	• intermediate professions	8.0	8.0	
	• farmers, employees, manual workers	6.9	6.9	
Alcohol consumption (g/d)		2.86	1.71	0.71
(medians [interquartile range])		[0.71–8.00]	[0–7.43]	
Smoking status (%)	Never smoked	55.2	49.4	0.41
	Former smoker	39.1	47.1	
	Current smoker	5.7	3.5	
Physical activity level (%)	High	37.9	28.7	0.14
	Moderate	33.9	28.7	
	Low	13.8	19.6	
	Unknown	14.4	23.0	
Season of inclusion (%)	Fall (Sept.–Nov.)	13.2	11.5	0.49
	Winter (Dec.–Feb.)	5.2	1.2	
	Spring (Mar.–May)	29.3	31	
	Summer (Jun.–Aug.)	52.3	56.3	
BMI (kg/m^2^)		23.76	25.58	0.006
(mean (SD))		(3.85)	(6.33)	
Corpulence class (%)	<25	67.8	59.8	0.003
	25–30	25.9	18.4	
	≥30	6.3	21.8	

* Matching values; ^1^ *p*-values based on paired comparisons using logistic models.

**Table 2:** Results from the different conditional logistic models showing a positive association between urinary 3-indoxylsulfate concentrations and probability of being a case (two CES-D scores ≥ 23; *n* = 87).

Model	Tertiles of log_10_ [3-Indoxylsulfate/Creatinine]			*p* for Trend ^7^
	T1 ^1^	T2	T3	
Model 1 ^2^	1	1.70 [0.88–3.28]	2.38 [1.24–4.58]	0.0088
Model 2 ^3^	1	1.73 [0.90–3.35]	2.47 [1.28–4.80]	0.0073
Model 3 ^4^	1	1.80 [0.90–3.58]	2.65 [1.31–5.35]	0.0066
Model 4 ^5^	1	1.70 [0.82–3.52]	2.52 [1.21–5.26]	0.0139
Model 5 ^6^	1	1.56 [0.72–3.38]	2.46 [1.11–5.45]	0.0264

^1^ Values are ORs [95% CI] for 2nd (T2) and 3rd (T3) tertiles of log_10_ [3-indoxylsulfate/creatinine] vs. 1st tertile (T1).^2^ for Model 1, the ORs were unadjusted.^3^ For Model 2, the ORs were adjusted for age.^4^ For Model 3, the ORs were adjusted for age, educational level, and marital status.^5^ For Model 4, the ORs were adjusted for age, education level, marital status, alcohol consumption, smoking status, physical activity level, and season of inclusion.^6^ For Model 5, the ORs were adjusted for age, educational level, marital status, alcohol consumption, smoking status, physical activity level, season of inclusion, and BMI. ^7^ *p*-values from logistic models with tertiles of log_10_ [3-indoxylsulfate/creatinine] as an ordinal variable in the model.

**Table 3:** Results from the different general linear models showing adjusted means [95% confidence interval] of log_10_ [3‑indoxylsulfate/creatinine] in the tertiles of fruit and vegetable intake in the study sample (*n* = 261).

Model	Tertiles of Fruit and Vegetable Intake			*p* for Trend
	T1	T2	T3	
Model 1 ^1^	2.34 [2.29–2.39]	2.37 [2.32–2.41]	2.24 [2.20–2.29]	0.0038
Model 2 ^2^	2.34 [2.30–2.39]	2.37 [2.32–2.41]	2.24 [2.19–2.28]	0.0021
Model 3 ^3^	2.35 [2.29–2.41]	2.38 [2.31–2.45]	2.25 [2.18–2.31]	0.0023
Model 4 ^4^	2.34 [2.25–2.42]	2.35 [2.26–2.44]	2.22 [2.14–2.31]	0.0012
Model 5 ^5^	2.33 [2.25–2.42]	2.35 [2.26–2.44]	2.22 [2.14–2.31]	0.0011

^1^ Crude model; ^2^ model adjusted for age; ^3^ model adjusted for age, educational level and marital status; ^4^ model adjusted for age, education level, marital status, alcohol consumption, smoking status, physical activity level, and season of inclusion; ^5^ model 5 adjusted for age, educational level, marital status, alcohol consumption, smoking status, physical activity level, season of inclusion, and BMI.

## Data Availability

Researchers of public institutions can submit a collaboration request including their institution and a brief description of their project to collaboration@etude-nutrinet-sante. All requests will be reviewed by the steering committee of the NutriNet-Santé study. A financial contribution may be requested. If the collaboration is accepted, a data access agreement will be necessary and appropriate authorizations from the competent administrative authorities may be needed. Under current regulations, no personal data will be accessible.
